# Distinct mechanisms of attentional suppression: exploration of trait factors underlying cued- and learned-suppression

**DOI:** 10.1186/s41235-024-00554-w

**Published:** 2024-05-01

**Authors:** Matthieu Chidharom, Nancy B. Carlisle

**Affiliations:** Department of Psychology, Lehigh University, 17 Memorial Drive, Bethlehem, PA 18015 USA

**Keywords:** Inattention, Visual working memory, Negative templates, Singleton suppression, ADHD

## Abstract

Attention allows us to focus on relevant information while ignoring distractions. Effective suppression of distracting information is crucial for efficient visual search. Recent studies have developed two paradigms to investigate attentional suppression: cued-suppression which is based on top-down control, and learned-suppression which is based on selection history. While both types of suppression reportedly engage proactive control, it remains unclear whether they rely on shared mechanisms. This study aimed to determine the relationship between cued- and learned-suppression. In a within-subjects design, 54 participants performed a cued-suppression task where pre-cues indicated upcoming target or distractor colors, and a learned-suppression task where a salient color distractor was present or absent. No significant correlation emerged between performance in the two tasks, suggesting distinct suppression mechanisms. Cued-suppression correlated with visual working memory capacity, indicating reliance on explicit control. In contrast, learned-suppression correlated with everyday distractibility, suggesting implicit control based on regularities. These results provide evidence for heterogeneous proactive control mechanisms underlying cued- and learned-suppression. While both engage inhibition, cued-suppression relies on deliberate top-down control modulated by working memory, whereas learned-suppression involves implicit suppression shaped by selection history and distractibility traits.

## Significance Statement

Everyday failures to filter out distractions, like when driving, can have severe real-world consequences. Elucidating the proactive cognitive control processes that allow us to anticipate and suppress distraction is thus critical. The field has developed two relevant experimental paradigms—cued-suppression and learned-suppression—that probe these anticipation-based filtering mechanisms. However, whether these tasks engage a common distraction suppression process remains unknown. Our study tackled this outstanding question by having participants complete both tasks and correlating performance. No relationship emerged, implying distinct underlying mechanisms. Further supporting this dissociation, the tasks related to distinct cognitive capacities. These findings advance theoretical models and pave the way for tailored interventions targeting real-world distraction filtering failures.

## Introduction

Distractions play a significant role in our daily lives, with consequences that can range from minor inconveniences to potentially serious outcomes. Consider a visual search task like looking for car keys on a cluttered desk, where a low consequence distraction can manifest as a notification appearing on your phone. This momentary diversion tempts you to check the message, momentarily shifting your attention away from the search. While it may slow down the key-finding process, its overall impact on the task remains relatively insignificant. However, distractions with high consequences become strikingly apparent when driving on a busy highway. A ringing phone or message notification can pull your focus away from the road, creating a serious risk. Succumbing to such distractions not only jeopardizes your safety but also poses a significant threat to the safety of others. We also know that difficulty with distractions can interfere with everyday functioning, as in individuals characterized by heightened distractibility, such as attention-deficit/hyperactivity disorder (ADHD) patients (Roy et al., 2020). Although the ability to suppress irrelevant items is thus crucial in our everyday life, we still lack a comprehensive understanding of how we effectively direct our attention away from distractors.

Current theories of visual attention provide valuable insights into our ability to orient attention toward targets. The Biased Competition Theory (Desimone, 1998; Desimone & Duncan, 1995), the Theory of Visual Attention (Bundesen, 1990), and the Guided Search Model (Wolfe et al., 1989) emphasize how visual working memory can automatically guide attention toward items that match its contents, facilitating efficient target selection. These theories focus on our ability to direct attention toward target items, but they neglect our ability to direct attention away from distractors. The importance of visual distractors on search efficiency has been long recognized. For example, Duncan and Humphreys (1989) demonstrate that lower feature similarity between targets and distractors improves search performance, indicating that dissimilar distractors are easier to distinguish from target and better suppress (Duncan & Humphreys, 1989). This work focused solely on how similar distractors are to the target, but did not focus on how attentional mechanisms might be altered by representations of distractor information itself.

However, a growing body of research highlights the crucial role of distractor processing for efficient visual search. Throughout our paper, we attempt to use definitions consistent with a recent consensus paper on terminology in attentional suppression (Liesefeld, et al., 2024).

Geng et al. (2017) manipulated the probability of target-similar distractors during a visual search task and measured the contents of the attentional template (Geng et al., 2017). They found that adjusting the contents of the attentional template can enhance visual search by increasing target-to-distractor distinctiveness and optimizing attentional selection. Geng et al. (2017) manipulated the probability of target-similar distractors during a visual search task and measured the contents of the attentional template, which is the mental representation guiding the search for relevant stimuli (Geng et al., 2017). They discovered that adjusting this template—by refining the mental representation to increase target-to-distractor distinctiveness—can enhance visual search performance through optimized attentional selection.

Similarly, prior experience with specific distractor values can lead to the development of relational templates which define the target in relation to distractors, instead of in absolute terms (Becker, 2010). Furthermore, Chetverikov et al. (2016) discovered that prior experience with distractors impacts visual search efficiency. They found that following a more diverse distractor distribution, observers took longer to locate a target, suggesting that the variability of distractor sets during visual search can influence our ability to efficiently detect targets (Chetverikov et al., 2016). Collectively, these recent findings underscore the importance of better characterizing distractor processing mechanisms to guide an efficient visual search.

To better understand our ability to guide attention away from distractors, recent studies developed two main attentional suppression paradigms: a *cued-suppression* and a *learned-suppression* task (see Geng et al., 2019, for a review). The *cued-suppression* task is based on a top-down control mechanism. In this task, attention is deliberately directed away from distractor items based on intentional instructions or goals. An illustrative example of this phenomenon is the study conducted by Arita et al. (2012). In their experiment, participants were tasked with searching for a shape-defined target among a display containing two colors of Landolt-Cs. Before each search display, a cue was presented, which could either provide information about the upcoming distractor color (negative cues), indicate the upcoming target color (positive cues), or be non-informative (neutral cues). The results of the study revealed that both negative and positive cues led to faster response times (RTs) compared to neutral cues. However, the benefits derived from negative cues were relatively smaller in magnitude when compared to positive cues (Arita et al., 2012). These findings have been replicated in subsequent studies (Addleman & Störmer, 2022; Carlisle & Nitka, 2019; Conci et al., 2019; Kerzel & Huynh Cong, 2022; Reeder et al., 2017, 2018; Zhang & Carlisle, 2022; Zhang et al., 2020). The replication of these benefits induced by negative templates suggests that attentional control can be configured to both direct attention toward target features and divert attention away from distractor features.

Recent studies have made significant strides in uncovering the mechanisms underlying cued-suppression and have revealed the role of a mechanism reported in the literature as proactive control. The proactive control mechanism is thought to enable the anticipation of distractor features and actively prevents attentional capture by irrelevant items. Notably, an fMRI study investigating *cued-suppression* demonstrated that, prior to visual search, negative cues elicited lower activation in early visual cortex regions compared to neutral cues, while positive cues led to increased activation in the same areas (Reeder et al., 2017). This finding suggests that negative templates guide attention by suppressing activity in visual areas associated with the processing of distractor features (Reeder et al., 2018). Moreover, EEG studies have also indicated heightened proactive engagement following negative cues, as evidenced by increased theta-band activity (4–8 Hz) over frontoparietal regions (Chidharom & Carlisle, 2023; de Vries et al., 2019). Theta-band activity is a well-documented brain oscillation associated with cognitive control (Cavanagh & Frank, 2014). Finally, by analyzing RT variability as a marker of proactive control efficiency, Chidharom and Carlisle (2024) recently revealed that individuals with greater ability to engage proactive control exhibit greater benefits after negative cues. A lower RT variability has indeed been associated with higher proactive engagement in both within (Chidharom & Bonnefond, 2023; Chidharom et al., 2021a, 2021b)- and between-subject design (Chidharom et al., 2021a, 2021b; Cooper et al., 2017; Mäki-Marttunen et al., 2018), suggesting a key role of preparation processes to exhibit consistent responses. Collectively, these findings underscore the crucial role of proactive control in effectively redirecting attention away from distractor items during cued-suppression.

Contrary to cued-suppression paradigm, the *learned-suppression task* relies on selection history (Awh et al., 2012; Geng & Behrmann, 2005) with distractors becoming easier to ignore after repeated exposure (Vatterott & Vecera, 2012; Vatterott et al., 2018; Won & Geng, 2020). For instance, Gaspelin and colleagues conducted a series of studies involving a visual search task where a uniquely colored distractor, such as a red singleton, appeared on the screen (Gaspelin & Luck, 2018a; Stilwell & Gaspelin, 2021; Stilwell et al., 2022). In earlier work, such singletons were thought to capture attention and interfere with target selection (Theeuwes, 1992). However, the authors demonstrated that if the color singleton consistently reoccurred as a distractor across multiple visual search trials, the negative impact on performance was reduced or could even lead to improved visual search performance (Gaspelin & Luck, 2018a; Stilwell & Gaspelin, 2021). This work builds upon a large prior literature examining the impact of salient distractors on attentional processing (e.g., Gaspar, et al., 2016; Gaspar & McDonald, 2014; Luck & Hillyard, 1994; Schubo & Muller, 2009; Wykowska & Schubo, 2011; Jannati, et al. 2013).

Interestingly, several studies have reported findings of proactive mechanisms underlying learned-suppression. One notable study by Gaspelin et al. (2015) employed an adapted probe method within a search task to investigate the suppression of salient distractors (Gaspelin et al., 2015). During search trials, participants were tasked with finding a specific target shape while disregarding a uniquely colored singleton distractor. Conversely, on random trials, letters briefly appeared at each search location before disappearing, and participants were required to report as many letters as possible. The results demonstrated that participants were less likely to report the letter at the singleton distractor location compared to the letters at non-singleton distractor locations. This probe suppression effect indicates that processing at the singleton location was actively inhibited, thereby impairing the encoding of the probe letter at that particular location. In other studies, researchers utilized eye-tracking to examine proactive guidance, specifically by measuring the first eye movement (Gaspelin & Luck, 2018b; Gaspelin et al., 2019). Interestingly, the gaze was *less* likely to be directed toward salient singleton distractors compared to average non-singleton distractor items, suggesting the existence of proactive oculomotor suppression. Furthermore, several EEG studies have investigated the P_d_ component, an event-related potential (ERP) believed to reflect the suppression of search items (Hickey et al., 2009; Sawaki et al., 2012). These studies, conducted by Gaspar and McDonald (2014), McDonald et al. (2013), and Kiss et al. (2012), consistently found that salient distractors elicited the P_d_ component, suggesting that singletons failed to capture attention and were actively suppressed (Gaspar & McDonald, 2014; Kiss et al., 2012; McDonald et al., 2013).

While both cued-suppression and learned-suppression are distinct areas of research that have been explored independently, an important question arises regarding the underlying mechanisms that support these two forms of suppression (Geng et al., 2019). It is noteworthy that a common proactive mechanism has been reported during both cued- and learned-suppression tasks, suggesting the possibility of shared mechanisms for both types of suppression. However, due to the separate exploration of these tasks in the literature, it remains challenging to arrive at a definitive conclusion. Therefore, the goal of this study is to investigate the extent to which cued-suppression and learned-suppression rely on similar control mechanisms. To accomplish this objective, we have employed two primary approaches. The first approach is a direct investigation (i.e., task to task), in which we will correlate the individuals’ performance in both cued-suppression and learned-suppression tasks. If cued-suppression and learned-suppression indeed rely on similar mechanisms, we would expect individuals who exhibit higher levels of suppression in one task to also demonstrate higher levels of suppression in the other task. The second approach is an indirect exploration (i.e., task-to-cognitive trait/function), in which we will correlate each task’s performance with different cognitive functions or traits, rather than directly with each other. We focused on the association between suppression performance and two factors: the individual’s visual working memory capacity (Poole & Kane, 2009) and their everyday life attentional suppression abilities, such as attentional distractibility. By investigating this association, we aim, once again, to determine whether attentional suppression effects rely on similar underlying mechanisms. If cued-suppression and learned-suppression share common mechanisms, we would anticipate both types of suppression to be associated with similar cognitive traits.

## Method

### Participants

The hypothesis, task design and statistical analysis plan were pre-registered on Open Science Framework (https://osf.io/rmfdn). Regarding our hypothesis on correlations, we anticipate discovering a moderate effect size of 0.45. This assumption is based on the previous studies discussed in the introduction, suggesting strong relationship between cued- and learned-suppression, as well as relationships with visual working memory and distractibility in everyday life thoughts. A power analysis thus suggested that 48 participants would be sufficient (by using G*Power 3.1, when setting alpha error = to 0.05 and 90% power, with two-tailed testing). We thus analyzed a large sample of 75 undergraduates from Lehigh University who gave informed consent and participated in a series of cognitive tasks for course credit (Mean Age = 19.11, SD = 1.15, 42 females). 21 participants were excluded for either bad accuracy in one of the cognitive tasks (n = 11) or for incomplete data recording (n = 10). Procedures were approved by Lehigh University IRB. All participants reported normal or corrected-to-normal vision and normal color perception.

### Procedure

Participants performed three different cognitive tasks for which the order was counterbalanced for every participant. Stimuli were presented using Psychtoolbox (Brainard & Vision, 1997) for MATLAB, and the screen was placed at a viewing distance of approximately 60 cm.

#### Cued-suppression task

**Stimuli**. Stimuli were presented on a gray background (90.0 cd/m^2^). Trials began with the presentation of a central fixation cross for 500 ms. A filled circle color cue (1.3°) was then presented at the center of the screen for 300 ms, followed by a 500 ms presentation of fixation point. Finally, the search items were presented until participants responded. If participants did not make a response within 3,500 ms, the trial was terminated. Search items were outlined circles (1.3° in diameter with a 0.2°line thickness) with a gap (0.5° long) that were presented 6.3° from fixation. The two colors appearing during the search array were randomly selected on each trial from a set of six colors (red, green, blue, magenta, orange, and cyan).

**Fig. 1:**
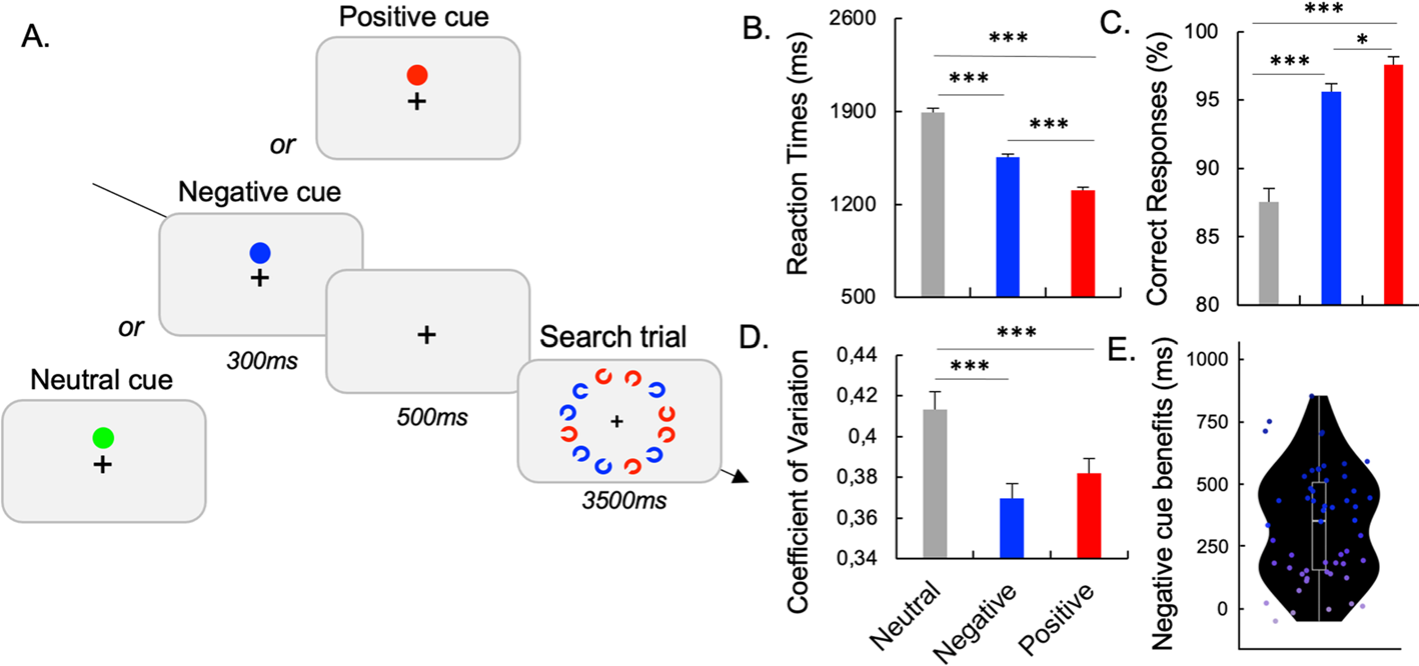
Cued-Suppression Task. In separate blocks, neutral, negative, or positive cues were presented with randomized color selection per trial. Participants had to locate the oriented Landolt-C targets with a gap at the top or the bottom (**A**). Difference between cue conditions has been observed on performance through reaction time (**B**), correct responses (**C**), reaction time variability (**D**). **E** Negative cue was associated with large interindividual difference in RT benefits. Points in blue indicated individuals with higher RT benefits. Error bars represent

**Task**. The trial commenced with displaying a fixation point for 500 ms. Subsequently, a colored circular cue appeared for 300 ms to indicate the upcoming target color in the visual search task. After presenting a fixation display for 500 ms again, an array of 12 Landolt-C stimuli was shown arranged on an implicit circle centered on the fixation cross. The Landolt-Cs were shown in a random set of 2 colors out of red, green, blue, magenta, orange and cyan, with 6 items of each color intermixed spatially. The participant’s task was to detect the target Landolt-C gap orientation being top or bottom. They responded to indicate target detection, upon which the search array was visible for either 200 ms post-response or maximally 3500 ms. The experiment comprised 3 blocks of 30 trials for each of 3 distinct cue conditions, separated by cue type explanations. The positive cue denoted the color of the target in the upcoming trial set. Using this, participants could selectively search through those 6 items. Similarly, the negative cue marked the distractor color to potentially ignore 6 non-targets. Finally, the neutral cue was an irrelevant color. The blocks were ordered randomly between participants, with instructions and practice for each block’s cue meaning provided beforehand (Fig. 1A).

#### Learned-suppression task

**Stimuli**. The design matched the prior work of Stilwell & Gaspelin, 2021. 10 shapes were arranged circularly with 3.1° eccentricity from the screen center on a black backdrop. The fixation cross in the middle comprised a 0.49°-diameter gray (30.0 cd/m2, x = 0.306, y = 0.320) circle with two intersecting 0.49° × 0.12° black lines and a central 0.12° gray dot. The search array contained one 1.3° × 1.3° diamond, one 1.3°-diameter circle, two 1.3° × 1.3° triangles, two 1.3° × 1.3° hexagons, two 1.6° × 1.0° ovals and two 1.3° × 1.3° crosses. These were rendered in either red (30.0 cd/m2, x = 0.627, y = 0.330) or green (30.0 cd/m2, x = 0.292, y = 0.631). The target shape (circle or diamond) and color (green or red) stayed fixed for each participant, fully counterbalanced. Half the trials had all items in the target color (singleton-absent). The other half had one randomly chosen item rendered in the non-target color as a singleton distractor. Each shape contained a small 0.2° × 0.2° black dot randomly on the left or right edge. The locations of the target and singleton distractor were random except they never overlapped.

**Fig. 2:**
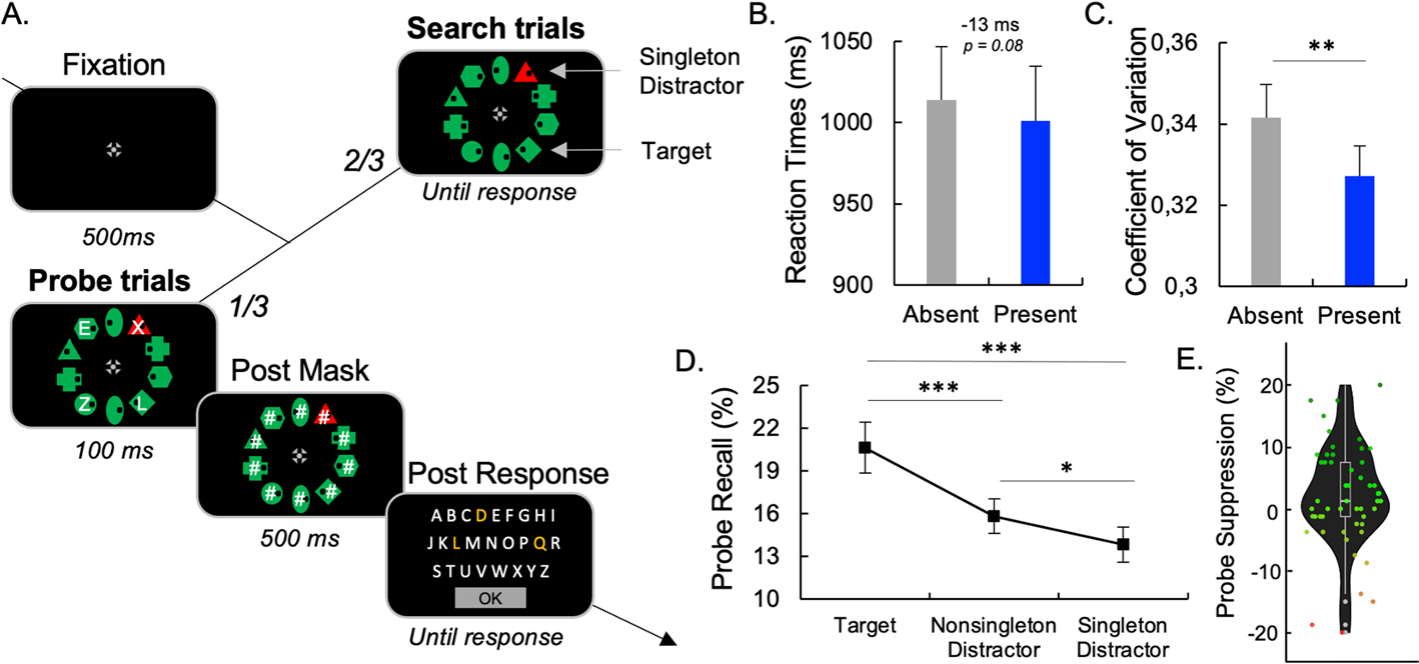
Learned-Suppression Task. **A** During search trials, individuals engaged in finding a specific target (e.g., a diamond) within an array of 10 different shapes. They had to swiftly press a button to show where a black dot was situated, either on the left or the right side. On probe trials, brief instances of white letters were overlaid onto the search objects. These letters were swiftly substituted with pattern masks (#). The participants’ objective was to recall and report as many of these letters as they could. **B** No difference in reaction times was observed when the singleton distractor was present vs. absent (**B**). C The reaction time variability was reduced when the singleton distractor was present vs. absent (**C**). **D** The probe recall is a function of the type of stimuli. **E**. Interindividual differences in the probe suppression effect (% non-singleton distractor *minus*. % Singleton distractor). In red, the participants reported more letters on singleton distractors, i.e., exhibiting attentional capture. Error bars represent standard errors. *p < 0.05. **p < 0.01; ***p < 0.001.standard errors. *p < 0.05; ***p < 0.001

**Task.** *On search trials*. On most trials the fixation appeared for 500 ms followed by the search array. Participants had to identify the dot location (left/right) within the target shape by clicking the respective mouse button, done as quickly as possible. Feedback on response time and accuracy was provided after every search. Error responses triggered a 300 ms, 200 Hz tone. Exceeding the 3000 ms response deadline displayed a “Too Slow” message with a 300 ms, 200 Hz tone. Participants were instructed to ignore any color singleton as it would never be the target (Fig. 2A).

***On probe trials***. On the remaining trials, the 100 ms probe array showing white capital letters in 0.7° × 0.7° Arial font superimposed the search items after 500 ms of fixation. These letters were random selections without replacement from A-Z. The probe array was followed by 500 ms post-masks as white number symbols before presenting the alphabet for untimed multiple-choice letter selection. One letter always overlaid the target shape. Another letter overlaid a singleton distractor, if present in that trial. The other letters appeared on randomly chosen non-singleton shapes. Without a singleton distractor, one letter marked the target and three letters appeared on random non-singleton shapes (Fig. 2A).

First, participants practiced the search task alone through 60 trials to familiarize themselves. Next was practice on 60 intermixed search and probe trials. Finally, there were 4 blocks of 60 randomized search and probe trials, totaling 240 trials. This comprised 80 probe trials with equal singleton-present and singleton-absent conditions. Feedback on mean response time and accuracy was provided block-wise.

#### Visual working memory task: the change localization task

**Stimuli.** The design was identical to the one used by Zhao et al., 2023. All stimuli were colored squares (0.5° x 0.5°). Color squares could appear anywhere within two circular areas from fixation (3.8° and 7°). Each square could appear in one of the nine distinct colors with no repetitions within any trial (red, green, blue, magenta, yellow, cyan, orange, white, black).

**Fig. 3:**
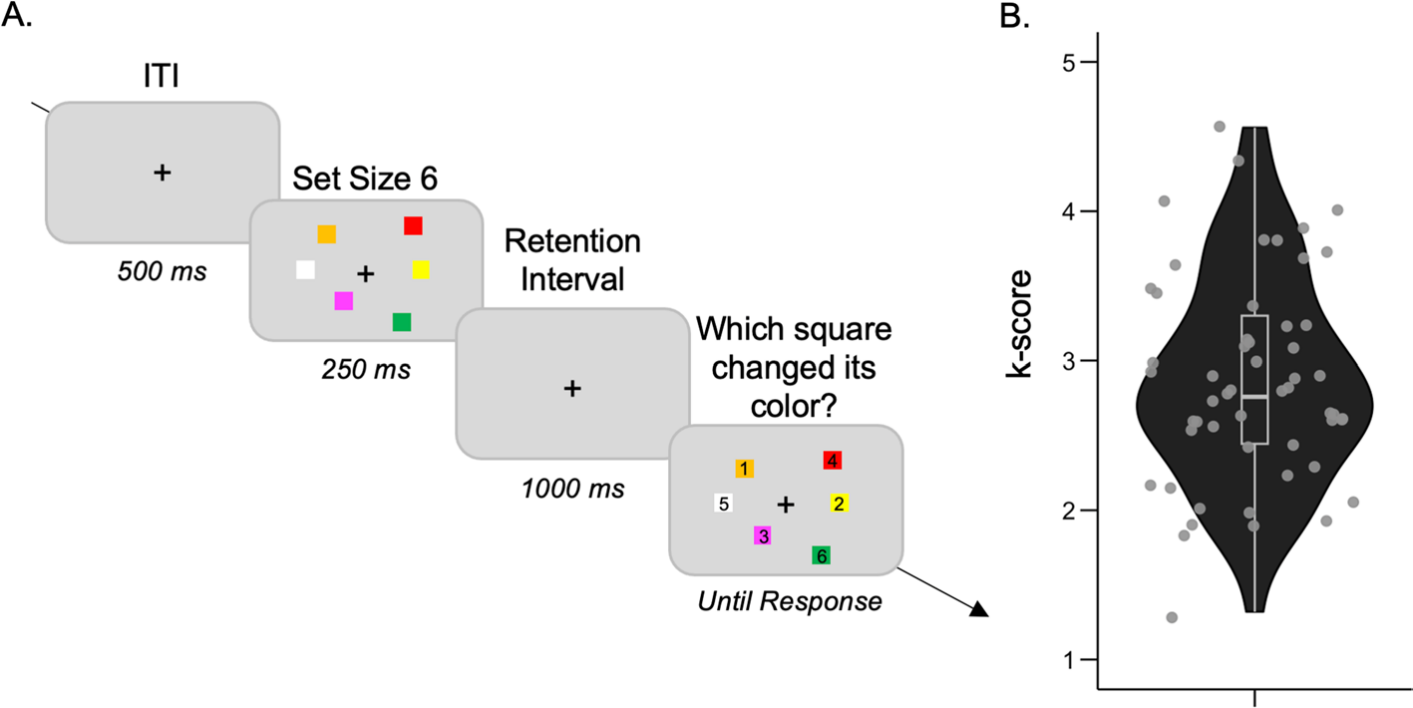
The change localization task. **A** Six colored squares emerged on the screen all at once. When the trial ended, participants were presented with the same six squares in the same positions as in the original previous trial. Among these squares, one would change its color, and participants were required to indicate the specific square that underwent this color change by using keyboard buttons. In the subsequent test phase, each square would display a number, prompting participants to press the corresponding button. **B** Interindividual differences in k score

**Task.** In each trial, six colored squares appeared simultaneously for 250 ms, followed by a 1,000-ms blank retention interval. Then, the six squares were presented again in the same locations, with one of the six colors changed to a color that had not been presented in the trial. Each square was marked with a digit (from 1 to 6), and participants pressed the corresponding key to indicate the item that had changed color. Responses were untimed and the spatial position of the six numbers was randomized across trials. Each participant completed 40 trials of the change localization task in total. Some practice trials were performed before starting the regular task (Fig. 3A).

#### Everyday life distractibility

**Fig. 4:**
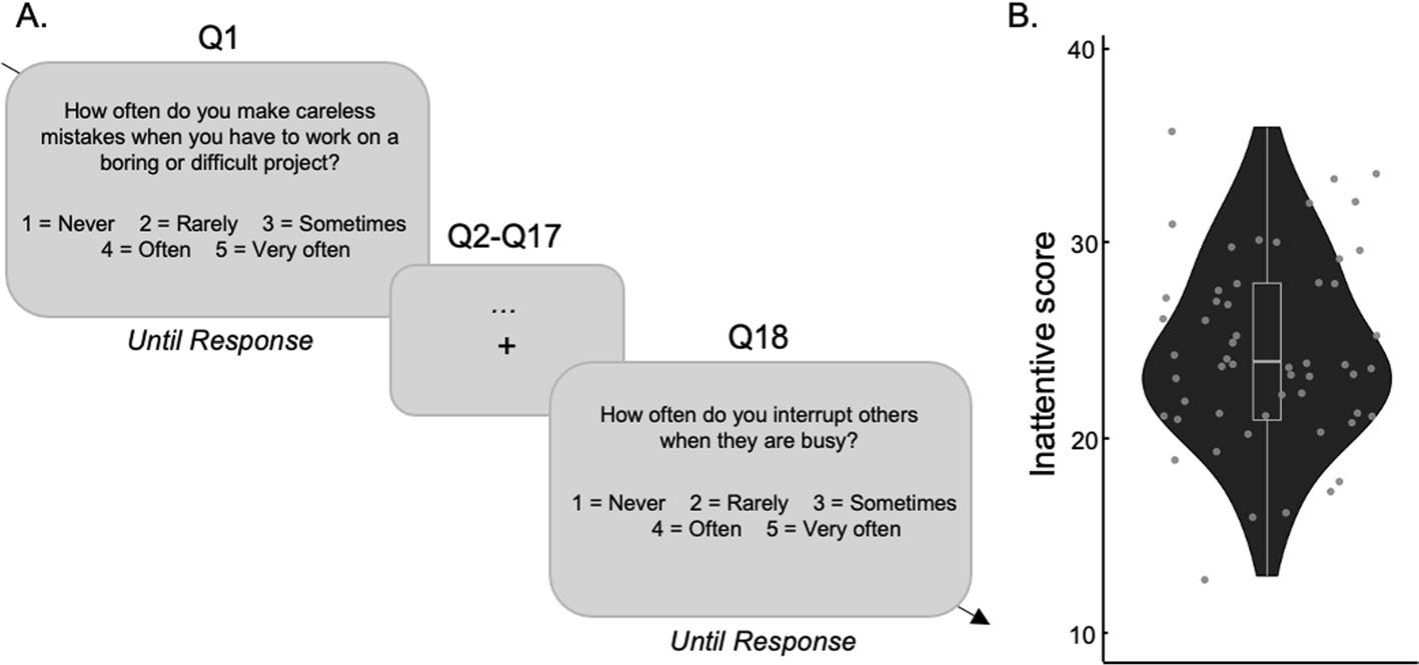
Adult ADHD Self-Report Scale. **A** Participants completed the questionnaire by using a 5-point Likert-type scale. **B** Interindividual differences in inattentive scores

Before performing these three cognitive tasks, all participants filled out the Adult ADHD Self-Report Scale (ASRS, Kessler et al., 2005), which enabled us to measure the everyday life distractibility of every participant. The scale contains the 18 symptoms of inattention, hyper-activity and impulsivity defining ADHD according to the DSM-IV-TR and DSM-5 (American Psychiatric Association, 2013). The severity of the symptoms is reported on a 5-point Likert-type scale (1–5 = never, rarely, sometimes, often, to very often) (Fig. 4A).

### Data and statistical analysis

For all the tasks, participants with 2.5 deviations from the mean accuracy have been excluded.

#### Cued-suppression task

For the cued-suppression task, trials with RT less than 300 ms or with incorrect responses were excluded from the analysis. The percentage of correct responses is the number of correct responses divided by the total number of trials. The variability of RT was explored through the coefficient of variation (CV) by dividing the standard deviation of the RT by the mean. To explore the effect of Cue on performance, ANOVAs including the within-subject factor Cue (Neutral, Negative and Positive) were performed. In the case of statistically significant interactions, paired *t tests* were conducted. Benefits in performance were measured through the difference between informative cues (positive and negative cues) and neutral cues.

#### Learned-suppression task

For the capture-probe task, trials with RT less than 200 ms or with incorrect responses were excluded from the analysis. The percentage of correct responses is the number of correct responses divided by the total number of trials. The RT variability was explored through the CV by dividing the standard deviation of the RT by the mean. On the search trials, performances have been analyzed through Student’s t tests, including the within-subject factor Singleton Distractor (singleton-present/singleton-absent). On the probe trials, the % of letter recall has been analyzed through a repeated measure ANOVAs including the within-subject factor Search item (Target/Non-singleton Distractor/Singleton Distractor). In the case of statistically significant interactions, paired t tests were conducted. For the search trials, the singleton presence effect (benefit or cost) will be measured through the difference between Absent and Present trials. For the probe trials, the probe suppression effect will be measured through the difference between the probe recall accuracy of the non-singleton distractor and the singleton distractor conditions.

#### Visual working memory task: change localization task

For the visual working memory task, k score was computed for each subject in line with Zhao et al. (2022): k = (Accuracy*N^2^ − N)/(N − 1), where the setsize N equals 6.

#### Adult self-report scale

Based on Stanton et al., 2018, we isolated a set of items in the ASRS to measure the inattentive and hyperactivity/impulsivity traits of each individual. A confirmatory factor analysis (CFA) with the factor Inattention (Q1, Q2, Q3, Q4, Q7, Q8, Q9, Q10, Q11) and the factor hyperactivity/impulsivity (Q5, Q6, Q12, Q13, Q14, Q15, Q16, Q17, Q18) was conducted to verify that a priori selected items reflect the same inattentive trait construct in the current dataset. The CFA revealed that the model did not fit the data correctly, as revealed by *X*^*2*^(134) = 198, p < 0.001; and the fit indices CFI = 0.74; RMSEA = 0.093. In the next step, Q10 was excluded from the analysis because of a bad estimate for the Inattentive traits factor. After exclusion, the model fit the data with *X*^*2*^(118) = 130, p = 0.213; and appropriate fit indices CFI = 0.94; RMSEA = 0.043.

#### Pearson correlations

To explore if the capacity to suppress cued distractors relies on the same mechanisms that the learned-suppression, Pearson correlation analysis will be performed between raw behavioral performance or indices (e.g., RT benefits and probe suppression) between the cued- and the learned-suppression task. In the same manner, to explore if the attentional suppression abilities (cued- and learned-suppression) were similarly associated with the visual working memory ability and the everyday life suppression ability, Pearson correlation has been used.

## Results

### Cued-suppression task

**Reaction Time.** The pattern of RT was similar compared to previous published studies with the slowest RTs in the neutral condition (1,890 ms), followed by the negative cue (1,550 ms) and the positive cue condition (1,310 ms). The ANOVA performed on RT revealed a significant main effect of Cue, F(2,106) = 220, p < 0.001, η^2^p = 0.81. Post hoc paired t tests revealed shorter RT for positive, t(53) = 18.9, p < 0.001, d = 2.57, and negative, t(53) = 11.1, p < 0.001, d = 1.51, compared to neutral cues. Participants were also slower in the negative cue than the positive cue conditions, t(53) = 11.5, p < 0.001, d = 1.56 (Fig. 1B).

**Accuracy**. The accuracy was high for all conditions, with the highest accuracy in the positive cue condition (97.6%) followed by the negative cue (95.6%) and the neutral cue (87.5%). The ANOVA performed on the % of correct responses revealed a significant main effect of Cue F(2,106) = 54, p < 0.001, η^2^p = 0.51. Post hoc paired t tests revealed higher % of correct responses for positive, t(53) =—8.69, p < 0.001, d =—1.18, and negative t(53) =—7.24, p < 0.001, d =—0.99, compared to neutral cue conditions. Participants were also less accurate in the negative cue than the positive cue conditions, t(53) = -2.61, p = 0.012, d =—0.36 (Fig. 1C).

**Coefficient of Variation**. The CV was higher for the neutral condition (0.413) followed by the positive (0.382) and negative cue condition (0.370). The ANOVA performed on the CV revealed a significant main effect of Cue, F(2,106) = 12.5, p < 0.001, η^2^p = 0.19. Post hoc paired t tests revealed low variability for positive, t(53) = 3.45, p = 0.001, d = 0.47, and negative, t(53) = 4.81, p < 0.001, d = 0.65, compared to neutral cue conditions. No differences were observed between positive and negative cue conditions (p = 0.17) (Fig. 1D).

### Learned-suppression task

#### Search trials

Mean RT was shorter on singleton-present trials (1,001 ms) compared to singleton-absent trials (1,014 ms). The Student’s t test revealed a tendency for shorter RT in the singleton-present condition compared to the singleton-absent condition, t(53) = 1.79, p = 0.080, d = 0.24 (Fig. 2B).

The mean correct responses were high in both singleton-present trials (97.6%) and singleton-absent trials (97.3%) with no statistical differences between the two conditions (p = 0.299).

The coefficient of variation was significantly lower for the singleton-present condition (0.327) compared to the singleton-absent condition (0.342), t(53) = 2.94, p = 0.005, d = 0.40 (Fig. 2C).

### Probe trials

For the singleton-absent condition, the rate of recalling letters was significantly higher when the letter was presented on the target (19.5%) than when the letters were presented on non-singleton items (15.6%), t(53) = 4.59, p < 0.001, d = 0.62.

In the singleton-present condition, the rate of recalling letters was highest when the letters were presented on the target (20.6%), followed by non-singleton items (15.8%) and the singleton item (13.8%). The distribution of the data did not conform to a normal distribution, leading us to employ nonparametric tests for analysis. Our nonparametric analyses revealed a significant main effect of search items (Friedman X^2^ = 15.5, df = 2, p < 0.001). Further nonparametric post hoc tests showed that the recall rate for target items was significantly higher than that for singleton items (W = 1073, df = 53, p < 0.001) and non-singleton items (W = 993, df = 53, p < 0.001). Additionally, the recall rate for singleton items was significantly lower than that for non-singleton items (W = 789, df = 53, p = 0.040) (Fig. 2D).

### Visual working memory task: the change localization task

Very similar to what Zhao et al. (2023) reported, the mean accuracy was 56% (9.6) much higher than the statistical chance level at 16.67%. The mean k score was 2.86 (0.70) (Fig. 3).

### Adult self-report scale

The mean total ASRS score was 34.3 (9.46) with a mean inattention score of 18.1 (5.40) (Fig. 4).

### Pearson correlations analysis

#### Relationship between cued- and learned-suppression

**Fig. 5:**
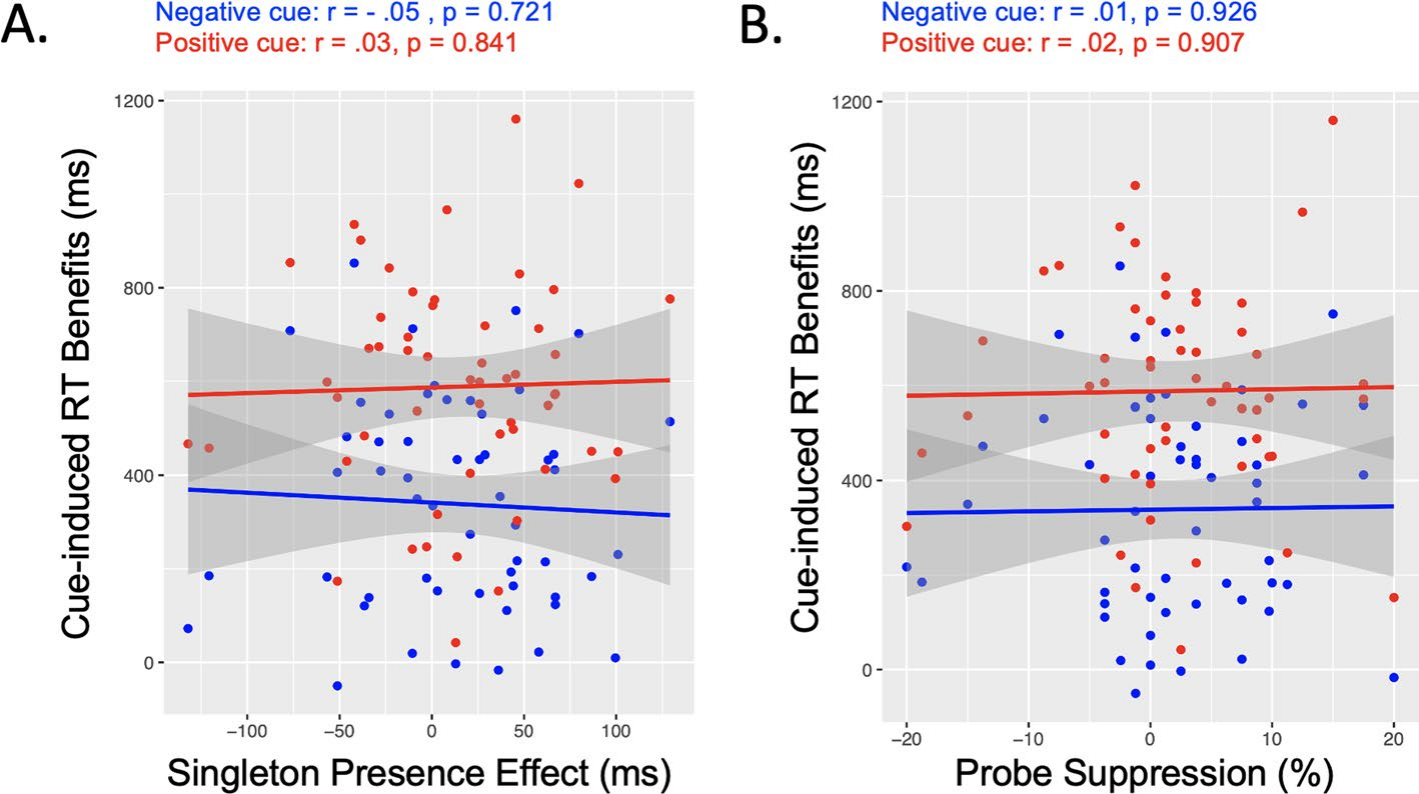
Relationship between cued- and learned-suppression task performance. The negative cue benefits were not associated with the learned-suppression, neither with the singleton presence effect (**A**) nor with the probe suppression (**B**)

On raw behavioral data, no significant relationship has been observed between the two tasks. Regarding suppression indices, the benefits on RT after both negative and positive cues of the cued-suppression task did not correlate with the singleton presence effect or the probe suppression effect of the learned-suppression task, all p values > 1 (Fig. 5).

#### Relationship between attentional suppression and visual working memory ability

**Fig. 6:**
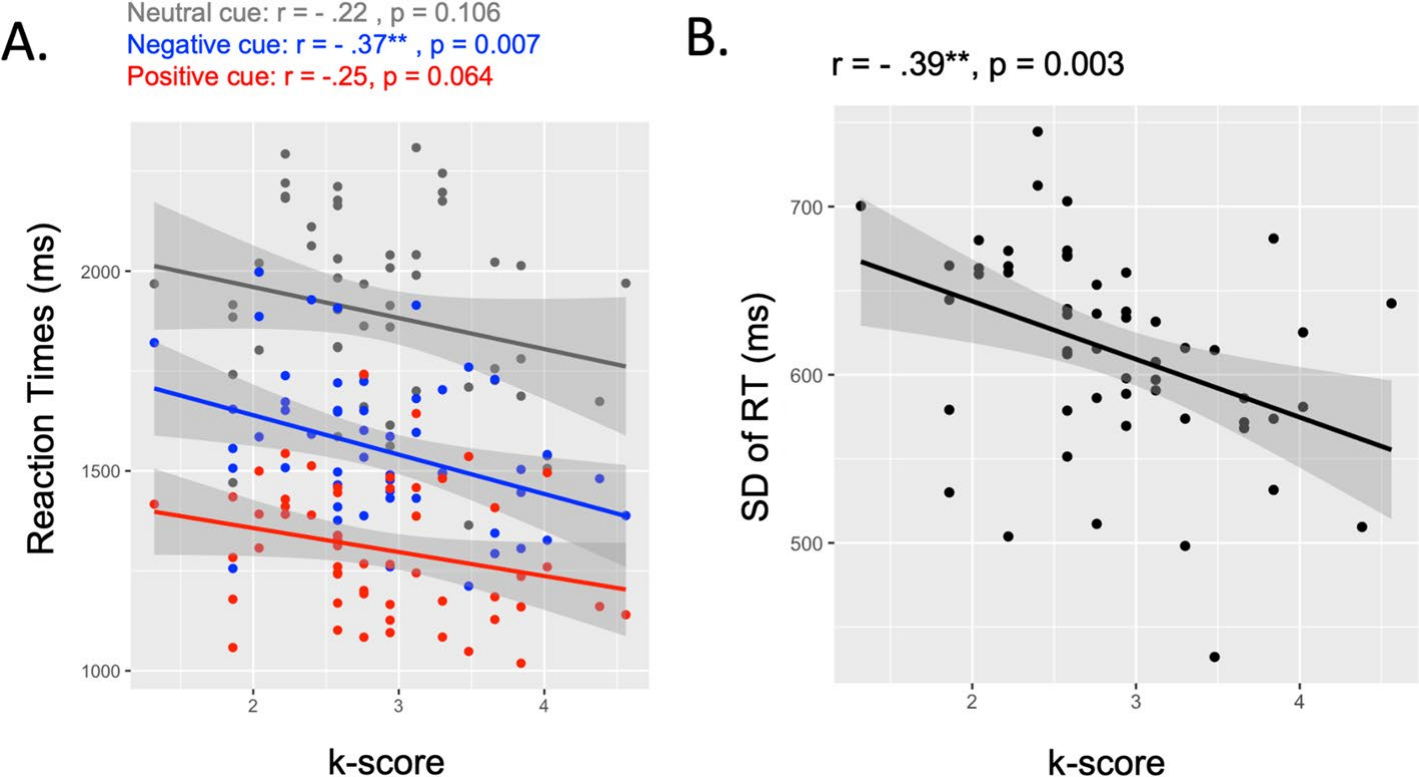
Relationship between the cued-suppression task performance and the VWM. The k score was specifically associated with performance of negative cue but not positive and neutral cue (**A**). The k score was associated with the RT variability (**B**). Individuals with lower RT variability exhibited higher k score. **p < 0.01

**Cued-suppression task.** Higher k score was associated with faster RT after negative cue, *r (54)* =—0.37, p = 0.007, but did not reach significance after positive (p = 0.064) and neutral cue (p = 0.106). Higher k score was also associated with higher accuracy in the neutral cue condition, *r* = 0.27, p = 0.045, but not in the negative (p = 0.375) and positive cue conditions (p = 0.896). Interestingly, the high WM ability was associated with a lower overall RT variability as revealed by the significant relationship between the k score and the SD-RT, *r(54)* =—0.39, p = 0.003 (Fig. 6).

**Learned-suppression task.** No relationship has been found with the visual working memory ability, all p values > 1 (Fig. 6).

#### Relationship between attentional suppression and everyday life suppression ability

**Fig. 7:**
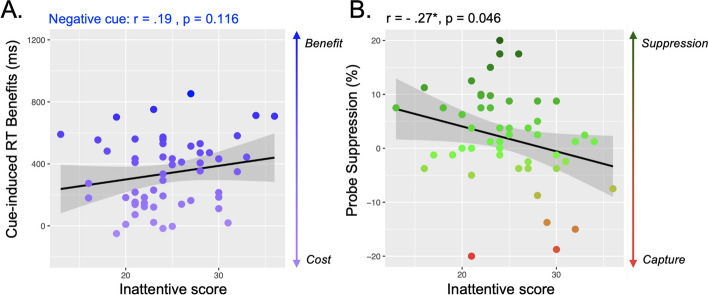
Relationship between the inattentive scores and suppression capacities. Inattentive score was not associated with the cued-suppression (**A**) but with the learned-suppression (**B**). *p < 0.05

**Cued-suppression task**. No relationship has been found between cued-suppression indices and inattentive trait scores, all p values > 1 (Fig. 7).

**Learned-Suppression task.** Regarding the raw performance, the Pearson correlation analysis revealed a negative correlation between the inattentive scores and the accuracy on the search trials when the singleton is absent, *r(54)* =—0.31, p = 0.021, and present, *r(54)* =—0.36, p = 0.007. Individuals with higher inattentive traits are less accurate during search. Moreover, the Pearson correlation analysis revealed a significant relationship between the probe suppression and the inattentive scores, *r*(*54*) =  − 0.27, p = 0.046; individuals with higher inattentive traits report more letters on singleton. No other relationship was found, all p > 1 (Fig. 7).

## Discussion

The goal of this study was to investigate the extent to which attentional suppression abilities rely on similar mechanisms to ignore irrelevant distractors. Specifically, we aimed to determine whether the control mechanisms underlying efficient performance in cued-suppression tasks are comparable to those in learned-suppression tasks. To address this question, we employed two distinct approaches. First, we conducted a correlational analysis to directly assess the relationship between performance in cued-suppression and learned-suppression tasks. Surprisingly, our results revealed no significant behavioral relationship between the two tasks, suggesting that the control mechanisms involved in facilitating effective performance in cued-suppression tasks are different from those in learned-suppression tasks. In order to further explore the underlying mechanisms, we adopted an indirect approach by examining the cognitive traits associated with suppression. Our findings demonstrated that cued-suppression was positively correlated with visual working memory capacity, whereas learned-suppression was found to be related to inattentive traits and everyday life distractibility. Overall, our study provides compelling evidence that different types of attentional suppression rely on distinct control mechanisms. The lack of a behavioral relationship between cued-suppression and learned-suppression tasks, combined with the differential cognitive traits associated with each type of suppression, underscores the heterogeneous nature of these processes.

### Evidence for a proactive mechanism during Cued- and Learned-suppression

Previous literature has consistently described a proactive control mechanism in both cued-suppression and learned-suppression tasks, enabling individuals to anticipate and efficiently redirect attention away from irrelevant distractors. In the current study, we aimed to further investigate the engagement of proactive control by examining reaction time (RT) variability as an index of its involvement, building upon prior research (Chidharom et al., 2021a, 2021b; Chidharom et al., 2021a, 2021b; Cooper et al., 2017). Our findings provide compelling evidence that both types of suppression rely on proactive mechanisms. Specifically, in the *cued-suppression task*, we observed significantly lower coefficient of variation after negative cues compared to neutral cues. These results align with previous studies and support the notion that negative templates rely on proactive control to effectively suppress distractors. Supporting neuroimaging evidence further substantiates this claim, as an fMRI study exploring cued-suppression revealed decreased activation in early visual cortex regions following negative cues (Reeder et al., 2017), suggesting that negative templates guide attention by suppressing activity in visual regions associated with distractor processing (Reeder et al., 2018). Additionally, EEG studies have consistently demonstrated heightened proactive engagement following negative cues, as indicated by increased theta-band activity (4–8 Hz) over frontoparietal regions (Chidharom & Carlisle, 2023; de Vries et al., 2019). Even more interestingly, a recent investigation by Chidharom & Carlisle (under review) unveiled that individuals with greater proactive control abilities exhibit greater benefits after negative cues, as reflected in interindividual differences in RT variability. Their analysis of the first eye movements revealed that individuals with superior proactive control were more adept at directing their attention away from distractor items.

Similar patterns of results emerged in the *learned-suppression task,* with a lower coefficient of variation observed when the singleton was present compared to absent. This finding is consistent with prior research that also highlighted proactive engagement to suppress salient singleton distractors. Notably, eye-tracking studies investigating proactive guidance demonstrated a reduced likelihood of gaze directed toward salient singleton distractors in comparison to average non-singleton distractors (Gaspelin & Luck, 2018a, 2018b; Gaspelin et al., 2019). Moreover, consistent findings from EEG studies indicated that salient distractors elicited the Pd component, suggesting that singletons failed to capture attention and were actively suppressed (Gaspar & McDonald, 2014; Kiss et al., 2012; McDonald et al., 2013). Collectively, our findings support the notion of proactive control engagement in both cued-suppression and learned-suppression tasks. The robust associations between RT variability, eye movements and neural activity provide converging evidence for the proactive mechanisms involved in suppressing distractors in these tasks.

### Opening the way to different proactive mechanisms underlie cued- and learned-suppression

Based on the existing literature and our current findings, we would anticipate that cued-suppression and learned-suppression rely on the same control mechanisms and exhibit shared variances. To directly investigate this hypothesis, we conducted the cued- and learned-suppression tasks within a single experiment. If these two types of suppression indeed rely on similar mechanisms, we would expect individuals who exhibit higher levels of suppression in one task to also demonstrate higher levels of suppression in the other task. However, our results did not support this assumption, as the correlational analysis between raw behavioral performance, negative cues benefits and probe suppression effect did not yield any significant relationships. These intriguing findings suggest the presence of distinct proactive control mechanisms for attentional suppression. We can speculate that cued-suppression predominantly relies on an *explicit proactive mechanism*. Indeed, the use of negative templates in cued-suppression tasks involves the engagement of top-down control mechanisms to actively suppress distractor features that are explicitly cued in advance. Participants are consciously aware of the distractor features they need to ignore and employ their attentional resources to suppress them. The increased theta activity following negative cues is in line with an effortful and voluntary top-down engagement over visual areas (Chidharom & Carlisle, 2023; de Vries et al., 2019). The cognitive effort required from negative templates could emerge from potential internal conflicts between the activation of working memory representations, which may naturally draw attention toward matching items, and the need to redirect attention away from these items.

Conversely, the learned-suppression mechanism may rely on an *implicit* form of proactive suppression that is probably less effortful compared to cued-suppression (Hauck et al., 2022). Indeed, learned-suppression is based on statistical regularities defining the distractors and is influenced by target-selection history and/or habituation (Geng et al., 2019). This learning process is thought to involve a reduction in the firing rate of neurons within the visual areas that encode prevalent but irrelevant sensory properties, effectively creating an internal model of what is unimportant within an environment and diminishing the orienting response to those stimuli (Bell et al., 2012; Rankin et al., 2009). Importantly, this learning process often occurs without conscious awareness of the regularities, indicating an implicit form of proactive control (Awh et al., 2012; Jiang & Sisk, 2019). Overall, our study provides evidence supporting the existence of different proactive control mechanisms for cued-suppression and learned-suppression. The lack of significant correlations and the distinct cognitive and neural profiles associated with each type of suppression suggest that explicit and implicit forms of proactive control may underlie these tasks.

The claim for existing different proactive control mechanisms of suppression is further supported by our complementary analysis that explores the cognitive factors associated with suppression. Indeed, we revealed that higher cued-suppression is associated with higher visual working memory capacity, whereas learned-suppression is rather correlated with lower inattentive traits.

### The visual working memory ability specifically associated with cued-suppression

The change localization task, which has been established as a novel and reliable measure of individuals’ visual working memory (VWM) capacities (Zhao et al., 2022), served as a valuable tool for investigating the relationship between VWM and cued-suppression in our study. Our results revealed a significant negative correlation between the k score (an indicator of VWM capacity) and RT following negative cues, but not RT following positive and neutral cues. This finding suggests that individuals with higher VWM abilities are more able to use negative cues to efficiently guide attention toward the critical target. This novel result adds to the existing literature, as previous research did not specifically associate VWM abilities with performance influenced by negative cues. Indeed, previous studies have demonstrated that negative templates are stored in VWM, as indicated by neurophysiological measures like the contralateral delay activity (CDA) observed in lateral occipital-temporal electrodes (Vogel & Machizawa, 2004; Vogel et al., 2005). Our study went further and highlighted the critical role of VWM capacities in effectively using negative cues. Additionally, we found that the k score was associated with overall RT variability. Specifically, individuals with higher standard deviation of RT (SD-RT) during the cued-suppression task exhibited lower VWM capacity. This finding aligns with previous research that emphasizes the crucial role of working memory in facilitating the efficient engagement of proactive control (Gonthier et al., 2019; Lin et al., 2022; Redick, 2014; Wiemers & Redick, 2018). For instance, Redick (2014) demonstrated that individuals with lower working memory capacity exhibit reduced engagement in proactive control compared to those with higher working memory capacity. Interestingly, our recent study also revealed that individuals with higher proactive control, characterized by lower SD-RT, experienced greater benefits from negative cues (Chidharom & Carlisle, under review). The current results suggest that individuals with lower SD-RT demonstrate higher working memory capacity (Buzy et al., 2009; Kofler et al., 2014; Moses et al., 2022), enabling them to effectively engage proactive control to suppress cued distractors. Overall, our findings highlight the critical role of VWM capacities in efficiently utilizing negative cues and suggest that individuals with higher working memory capacity demonstrate enhanced engagement of proactive control to suppress distractors.

### Learned-suppression associated with everyday life distractibility

Contrary to the performance observed in the cued-suppression task, we did not observe any relationship between the VWM capacity and the learned-suppression. This absence of result is in line with the recent paper of Hauck et al. (2022). Indeed, the authors established two categories of individuals with high and low visual working memory (VWM) and demonstrated a comparable learned-suppression ability within both groups (Hauck et al., 2022). However, this does not necessarily imply that the suppression of salient distractors is universally independent of VWM. For instance, Gaspar et al. (2016) observed a correlation between working memory performance and the ability to suppress singletons. In their task, the singleton can appear in two colors across the search trials (rather than just one in the current study), making the appearance of the distractors more variable and unpredictable. In addition, the Gaspar study had participants look for a color singleton target, while examining whether a secondary color singleton would capture attention. Therefore the Gaspar, et al. (2016) study differed from our own in terms in multiple respects. First, only a single distractor color needed to be ignored in our design, as is typical in learned-suppression studies (Gaspelin & Luck, 2018b), vs. needing to ignore multiple colors in Gaspar and et al., (2016). Secondly, participants were presumably in feature search mode in our study, while they may have been using singleton search in Gaspar and et al., (2016; see Bacon & Egeth, 1994). This may indicate that while learned-suppression, as we defined it, is not directly related to VWM, other broader forms of salient distractor suppression could still be associated with VWM capacities. Despite an absence of relation with VWM, we discovered a significant correlation between the inattentive trait measure and the performance of learned-suppression. Specifically, our findings unveiled a noteworthy association between scores on the inattentive trait scale of the ASRS and the probe suppression effect. This suggests that individuals with higher levels of daily distractibility tend to report more letters on the singleton compared to those with lower distractibility. These results align with previous literature focusing on ADHD patients. For instance, in a similar task, Zhang et al. (preprint) revealed that individuals with ADHD tend to fixate on distractors for longer durations compared to those without ADHD (H. Zhang et al., 2023). Similarly, Wang et al. (2016) reported that the ADHD group exhibited a significantly smaller PD component amplitude in response to singletons, indicating a deficiency in actively suppressing distractors among patients (Wang et al., 2016). Collectively, these findings imply a continuum in our ability to effectively suppress singleton distractors, varying between individuals with low and high levels of inattentive traits.

## Limitations

One limitation of our study is the lack of additional measures of other cognitive traits that could be associated with suppression performance. Although the VWM task and the measures of inattentive traits have been selected a priori, several other factors could be associated with suppression, such as anxiety traits (Gaspar & McDonald, 2018), and should be explored in further studies. Another limitation is the reliability of our suppression measures. Indeed, Hedge et al. (2018) reported that robust cognitive tasks did not always produce reliable individual differences (Hedge et al., 2018). However, the authors proposed some recommendations that fit with the current study. First, both cued- and learned-suppression tasks lead to inter-subject variability rather than homogenous performance, with some individuals exhibiting benefits and others experiencing costs during suppression conditions. Second, the use of multiple approaches (direct and indirect) allowed us to conclude differences in suppression mechanisms, with the change localization task (Zhao et al., 2022) and the ASRS showing high reliability (Green et al., 2019; Silverstein et al., 2018). Finally, the increased statistical power afforded by our large sample size allowed us to minimize the impact of measurement error. Future research should try to more directly establish the mechanisms underlying both cued- and learned-suppression, utilizing a combination of behavior and neuroscience techniques.

## Conclusions

In conclusion, our study elucidates the distinct cognitive control processes allowing anticipation and suppression of distraction, with profound real-world implications. The lack of relationship between cued- and learned-suppression performance, despite their superficial similarity, implies heterogeneous underlying mechanisms. These challenge assumptions of a unitary proactive filtering system. Furthermore, our results demonstrate that cued-suppression is associated with higher visual working memory capacity, highlighting its importance in effectively utilizing negative cues. In contrast, learned-suppression is related to inattentive traits and everyday life distractibility, suggesting a continuum in the ability to suppress singleton distractors based on individual traits. Elucidating suppression is an urgent, use-inspired goal; our results constitute a vital step forward.

## Data Availability

The codes and datasets generated and/or analyzed during the current study are fully available from the corresponding author on request.

## Abbreviations

RT: Reaction time
CV: Coefficient of variation
ANOVA: Analysis of variance
VWM: Visual working memory
CDA: Contralateral delay activity
EEG: Electroencephalography
fMRI: Functional magnetic resonance imaging
ERP: Event-related potential
ADHD: Attention-deficit/hyperactivity disorder
ASRS: Adult ADHD self-report scale
CFA: Confirmatory factor analysis
SD-RT: Standard deviation of reaction time
